# Analysis of time-domain indices, frequency domain measures of heart rate variability derived from ECG waveform and pulse-wave-related HRV among overweight individuals: an observational study

**DOI:** 10.12688/f1000research.139283.1

**Published:** 2023-09-27

**Authors:** Sinha Mukesh Kumar, K. Vaishali, G. Arun Maiya, K.N. Shivashankar, U. Shashikiran

**Affiliations:** 1 Department of Physiotherapy, Manipal College of Health Professions, Manipal Academy of Higher Education, Manipal, Karnataka, 576104, India; 2 Department of Medicine, Kasturba Medical college, Manipal Academy of Higher Education, Manipal, Karnataka, 576104, India; 3 Department of Medicine, Dr. TMA Pai Hospital, Udupi, Manipal Academy of Higher Education, Manipal, Karnataka, 576104, India

**Keywords:** Pulse Signal, Electrocardiogram, pulse rate variability, Adiposity, Cardiac autonomic function, sympathetic nervous system, parasympathetic nervous system

## Abstract

**Background:** Research on the compatibility of time domain indices, frequency domain measurements of heart rate variability obtained from electrocardiogram (ECG) waveforms, and pulse wave signal (pulse rate variability; PRV) features is ongoing. The promising marker of cardiac autonomic function is heart rate variability. Recent research has looked at various other physiological markers, leading to the emergence of pulse rate variability. The pulse wave signal can be studied for variations to understand better changes in arterial stiffness and compliance, which are key indicators of cardiovascular health.

**Methods:** 35 healthy overweight people were included. The Lead II electrocardiogram (ECG) signal was transmitted through an analog-to-digital converter (PowerLab 8/35 software, AD Instruments Pty. Ltd., New South Wales, Australia). This signal was utilized to compute Heart Rate Variability (HRV) and was sampled at a rate of 1024 Hz. The same AD equipment was also used to capture a pulse signal simultaneously. The right index finger was used as the recording site for the pulse signal using photoplethysmography (PPG) technology.

**Results:** The participants’ demographic data show that the mean age was 23.14 ± 5.27 years, the mean weight was 73.68 ± 7.40 kg, the mean body fat percentage was 32.23 ± 5.30, and the mean visceral fat percentage was 4.60 ± 2.0. The findings revealed no noticeable difference between the median values of heart rate variability (HRV) and PRV. Additionally, a strong correlation was observed between HRV and PRV. However, poor agreement was observed in the measurement of PRV and HRV.

**Conclusion:** All indices of HRV showed a greater correlation with PRV. However, the level of agreement between HRV and PRV measurement was poor. Hence, HRV cannot be replaced with PRV and vice-versa.

## Introduction

Assessing the cardiac autonomic nervous system’s functioning, heart rate variability (HRV) obtained from electrocardiographic (ECG) signals is a useful tool. Over the past decade, HRV measures have been used in a variety of clinical contexts as indicators of sympathovagal interaction and to diagnose various clinical and functional conditions. ^1^^,^^2^ Analysis of the time and frequency domains can be used to quantify heart rate variability. ^1^ Statistics such as mean heart rate, SDNN (standard deviation of normal-to-normal intervals), RMSSD (root mean square of successive differences), and pNN50 (percentage of successive normal-to-normal intervals that differ by more than 50 millisecond) are among the time domain measures of HRV. The primary time-domain measure used to calculate the vagally mediated changes indicated in HRV is the RMSSD, which reflects the beat-to-beat variance in HR. ^1^^,^^3^ The pNN50 is closely correlated with peripheral nervous system activity and it is correlated with the RMSSD and high frequency (HF) power. Frequency domain of HRV is measured using spectrum analysis of ECG signal. High frequency (HF) and low frequency (LF) are the two primary sub-bands of the HRV frequency spectrum. ^1^^,^^3^ HF power reflects parasympathetic activity, whereas LF power may indicate both sympathetic and parasympathetic activity. ^1^^,^^3^ The LF/HF power ratio is often employed as a measure of sympathovagal balance and total power (TP) represents overall variability. ^1^^,^^3^^,^^4^

Atherosclerosis development, myocardial infarction, and heart failure have all been associated with low HRV indices. Studies have shown that the reduced HRV value is also associated with conditions like coronary artery disease, diabetes mellitus, pain, acute and chronic stress, metabolic syndrome, hypertension, and physical inactivity. ^5^^–^^7^ An autonomic nervous system that is more adaptable and resilient is indicated by a higher HRV. ^3^ Also the use of HRV as a social interaction, athletic performance, and emotional state marker is also common. ^8^^,^^9^

The ECG signal is frequently used to estimate HRV. The use of physiological signals other than the ECG to produce HRV data has been established in a number of research during the past few years. ^10^ The data on HRV derived from pulse wave signals; thus it is referred to pulse-wave-related HRV or pulse rate variability (PRV). ^10^^–^^12^ Variations in the pulse wave pressure are indicative of changes in arterial stiffness and compliance, two pivotal facets of cardiovascular well-being. ^13^ Elevated arterial stiffness, commonly observed in cardiovascular disorders, can precipitate modifications in the pulse wave pressure, underscoring its importance in assessing cardiovascular health. ^13^^–^^15^

The term compatibility between PRV and HRV characteristics is a subject of interest for current research, due to its frequent use in clinical and everyday situations, researchers have tried to extract as much information as possible from pulse signals to understand the similarity between PRV and HRV characteristics. ^10^^,^^16^ For instance, the findings from a study by Wong JS ^17^ showed that the time-varying spectral indices generated from HRV and PRV have a few minor differences. This suggests that PRV can be considered as an alternate measurement to HRV. When they compared the results from the healthy subject during regular breathing, they came to similar conclusions. According to the study cited by Ahsan H. Khandoker, ^18^ PRV does not accurately reflect HRV in individuals with obstructive sleep apnea. The accuracy of PRV as a substitute for HRV has recently been the subject of numerous studies, but it has not been examined in overweight individuals, who appear to have lower heart rate variability than healthy normal individuals. The purpose of this study was to determine whether healthy overweight individuals’ PRV can serve as a substitute for HRV.

## Methods

### Study design and settings

A study was conducted in the Cardiopulmonary Physiotherapy Laboratory at Kasturba Hospital in Manipal, Karnataka, India. This is a cross-sectional study, which is part of a large intervention trial. The study protocol was approved by the Institutional Research Committee (IRC), and Institutional Ethics Committee of Kasturba Medical College and Kasturba Hospital Manipal (IEC/155/2018). The trial has been registered under the Clinical Trials Registry (CTRI number: CTRI/2018/08/015523).

### Participant recruitment and selection criteria

This study followed the Strengthening the Reporting of Observational Studies in Epidemiology (STROBE) guidelines. This study included 35 healthy overweight individuals (20 women and 15 men, aged 23.13 ± 5.27 years). Upon verbal advertising, willing individuals were screened for eligibility. Each participant signed a written informed consent form before the study’s process started. Participants received detailed information about the study’s objectives, procedures, and rights to withdraw at any time. Participation in the study was entirely voluntary. Prior to the HRV recording, participants were asked to abstain from drinking anything alcoholic or caffeinated for at least 12 hours.

### Eligibility criteria

The study enrolled participants with any genders, aged between 18 and 40 years, with a body mass index (BMI) ranging from 25 to 29.9 kg/m ^2^, and assessed to have low risk based on the AHA/ACSM Health/Fitness Facility Preparticipation Screening Questionnaire. ^19^ The study excluded participants who had been diagnosed with cardiopulmonary disease, diabetes mellitus, hypertension, metabolic disorder or were taking any regular prescribed medication for renal and liver disease.

### Measurement of heart rate variability and pulse rate variability

The participants were instructed to avoid consuming caffeine, alcohol, or other stimulants for at least 12 hours before the test. They were also advised to avoid any strenuous activity for at least 2 hours before the test. A properly calibrated instrument from AD Instruments Pty. Ltd. (New South Wales, Australia) was used to record the ECG signal. Electrodes were placed on the subject’s chest to record the ECG signal. The participants were asked to lie down in a quiet, comfortable room (temp- 22-24 ^o^C was maintained during HRV recording). The ECG signal was recorded for 15 minutes while the subject was at rest. The recorded ECG signal was processed using software PowerLab 8/35 in LabChart (AD Instruments, PowerLab 8/35 on LabChart for Windows version) that is designed to analyze HRV. Also simultaneously pulse signal was recorded with the help of same AD Lab instrument (AD Instruments Pty. Ltd.) and signal acquisition was obtained on the AD instrument. The pulse signal was recorded using photoplethysmography (PPG) from the right index finger. The recorded pulse signal was processed using the PowerLab 8/35 software to synchronise the event in LabChart (Alternatively, Kubios HRV Standard software can also be used for HRV analysis). The analysis involves identifying the time domain and frequency domain measures of PRV. Time domain analysis includes SDNN (Standard deviation of NN intervals), RMSSD (Root mean square of successive RR interval differences), and pNN50 (Percentage of successive RR intervals that differ by more than 50 ms). The frequency domain analysis includes High frequency (HF), low frequency (LF), LF/HF ratio, and total power.

### Measurement of cardiorespiratory fitness (CRF)

All participants performed the cardiopulmonary exercise test using the Bruce protocol. ^19^ Participants were screened for eligibility and informed of the testing procedures. A heart rate monitor (Polar FT1 heart rate monitor watch with polar T31- CODED transmitter) and a blood pressure cuff (Diamond Dial Deluxe Blood Pressure Apparatus) were connected to the patient to record the vitals. The participants were asked to warm up for 2-3 minutes by walking on a treadmill (Runner 7410, Runner Srl, Cavezzo, Italy) at a slow pace, with a 0% grade. The test was started with the treadmill set at a 10% grade and a speed of 2.7 km/h. The participants were asked to walk at this pace for 3 minutes. Thereafter every 3 minutes’ treadmill speed and inclination was increased as per standard Bruce protocol. ^19^ Exercise test termination was considering as per standard recommendation. ^19^ The test was terminated if the individual reached volitional fatigue or about 85% of age-predicted HRmax, displayed adverse signs or symptoms, requested to stop. An adequate cool-down/recovery interval was provided in the form of continuous walking at a work rate equal to that of the first stage of the exercise test protocol or lower. ^19^ Throughout the test, heart rate, blood pressure, and Spo _2_ was monitored and recorded. The participants were also asked to rate their perceived exertion at the end of each stage using the Borg Rating of Perceived Exertion scale. ^19^ No adverse event was noted during or following the exercise test.

### Statistical analysis

Statistical Package for the Social Sciences (SPSS) version 15.0 was used to analyse the data (SPSS for Windows, Version 15, SPSS, Inc., Chicago, IL, USA). Also Jamovi software ^20^ was used for Bland-Altman analysis. Descriptive statistics was used to summarize the data. Measures such as mean, standard deviation, median, minimum, maximum, and range were calculated for HRV and PRV data separately. Correlation analysis was used to examine the relationship between HRV and PRV. Spearman rank correlation coefficient was used as data was not normality distributed. Mann-Whitney U test was performed to determine difference between HRV and PRV. Also, Bland-Altman analysis was carried out between HRV and PRV for level of agreement in measurement.

## Results

A total of 35 participants (20 females and 15 males) data were analyzed in this study. Table 1 describes the demographic details of the participants, where the mean age was 23.14 ± 5.27 years, weight was 73.68 ± 7.40 kg, body fat % was 32.23 ± 5.30 and visceral fat % was 4.60 ± 2.0. Table 2 describes comparison of time domain indices, frequency domain measures (absolute parameter) of HRV and PRV of the participants. The Mann Whitney U test was performed to compare HRV and PRV for absolute parameter, the results showed no significant difference between the median HRV and PRV values (p-value ≥ 0.05).

**Table 1.** Demographic details of the participants (N = 35).

Variables:	Mean ± S.D
Age (Years)	23.14 ± 5.27
Height (cm)	163.56 + 6.41
Weight (kg)	73.68 ± 7.40
BMI (kg/m ^2^)	27.50 ± 1.67
Body fat%	32.23 ± 5.30
Visceral fat%	4.60 ± 2.0
RHR (bpm)	76 ± 5
Bruce protocol test time (minutes)	10.51 ± 1.29
VO _2_ Peak (mL/kg/min)	36.28 ± 5.34
HR Max (bpm)	181± 10

BMI - body mass index; RHR - resting heart rate; VO _2_ peak - peak oxygen uptake; HR Max - heart rate maximum.

**Table 2.** Comparison of time domain indices, frequency domain measures (absolute parameter) of heart rate variability and pulse rate variability of the participants.

Time domain and frequency domain variables	Heart rate variability (HRV)	Pulse rate variability (PRV)	p value
	Absolute values Median (interquartile range IQR)	Absolute values Median (interquartile range IQR)	
SDNN	56.6 (42.6, 77.0)	56.3 (40.3, 67.4)	0.94
RMSSD	46.4 (30.3, 59.7)	45.4 (34.0, 58.1)	0.52
pNN50	25.9 (7.63, 38.4)	24.7 (9.30, 37.5)	0.46
Total power	2367 (1814, 4724)	3865 (1845, 6762)	0.89
LF (ms ^2^)	586 (369, 1082)	964 (444, 1530)	0.61
HF (ms ^2^)	769 (379, 1501)	1219 (739, 2399)	0.31
LF/HF ratio	0.72 (0.48, 1.21)	0.54 (0.39, 1.13)	0.92

**Time-domain variables of HRV/PRV**: SDNN- Standard deviation of NN intervals (ms), RMSSD- Root mean square of successive RR interval differences (ms), pNN50 - Percentage of successive RR intervals that differ by more than 50 ms (%). **Frequency domain variables of HRV/PRV**: LF - Power in the low-frequency range (0.04–0.15 Hz), HF - Power in the high-frequency range (0.15–0.4 Hz), LF/HF ratio - Ratio of LF [ms ^2^]/HF [ms ^2^], TP - Total Power.

Table 3 describes comparison of time domain indices, frequency domain measures (Log transformed parameter) of HRV and PRV of the participants. A t-test was performed to compare HRV and PRV. The results showed no significant difference between the mean HRV and PRV values (p-value ≥ 0.05). Table 4 depicts Bland-Altman plot of Total Power measures of HRV and PRV.

**Table 3.** Comparison of time domain indices, frequency domain measures (log transformed parameter) of heart rate variability and pulse rate variability of the participants.

Time domain and frequency domain variables	Heart rate variability	Pulse rate variability	p value
	Log transformed mean ± SD/median [IQR]	Log transformed mean ± SD/median [IQR]	
SDNN	1.74 ± 0.15	1.71 ± 0.15	0.87
RMSSD	1.63 ± 0.19	1.65 ± 0.17	0.62
pNN50	1.20 ± 0.52	1.28 ± 0.39	0.74
Total power	3.37 ± 0.33	3.50 ± 0.35	0.85
LF (ms ^2^)	2.77 ± 0.34	2.88 ± 0.39	0.90
HF (ms ^2^)	2.89 ± 0.38	3.10 ± 0.38	0.95
LF/HF ratio	0.26 (0.4,0.05)	0.26 (0.4, 0.05)	0.96

**Time-domain variables of HRV/PRV**: SDNN - Standard deviation of NN intervals (ms), RMSSD - Root mean square of successive RR interval differences (ms), pNN50 - Percentage of successive RR intervals that differ by more than 50 ms (%).

**Frequency domain variables of HRV/PRV**: LF - Power in the low-frequency range (0.04–0.15 Hz), HF - Power in the high-frequency range (0.15–0.4 Hz), LF/HF ratio - Ratio of LF [ms ^2^]/HF [ms ^2^], TP - Total Power.

**Table 4.** Bland-Altman plot of Total Power measures of HRV and PRV.

	95% Confidence Interval		
	Estimate	Lower	Upper
Bias (n = 35)	930	373	1487
Lower limit of agreement	-2248	-3209	-1287
Upper limit of agreement	4108	3147	5069

HRV - Heart rate variability; PRV - Pulse rate variability.

**Figure 1.**
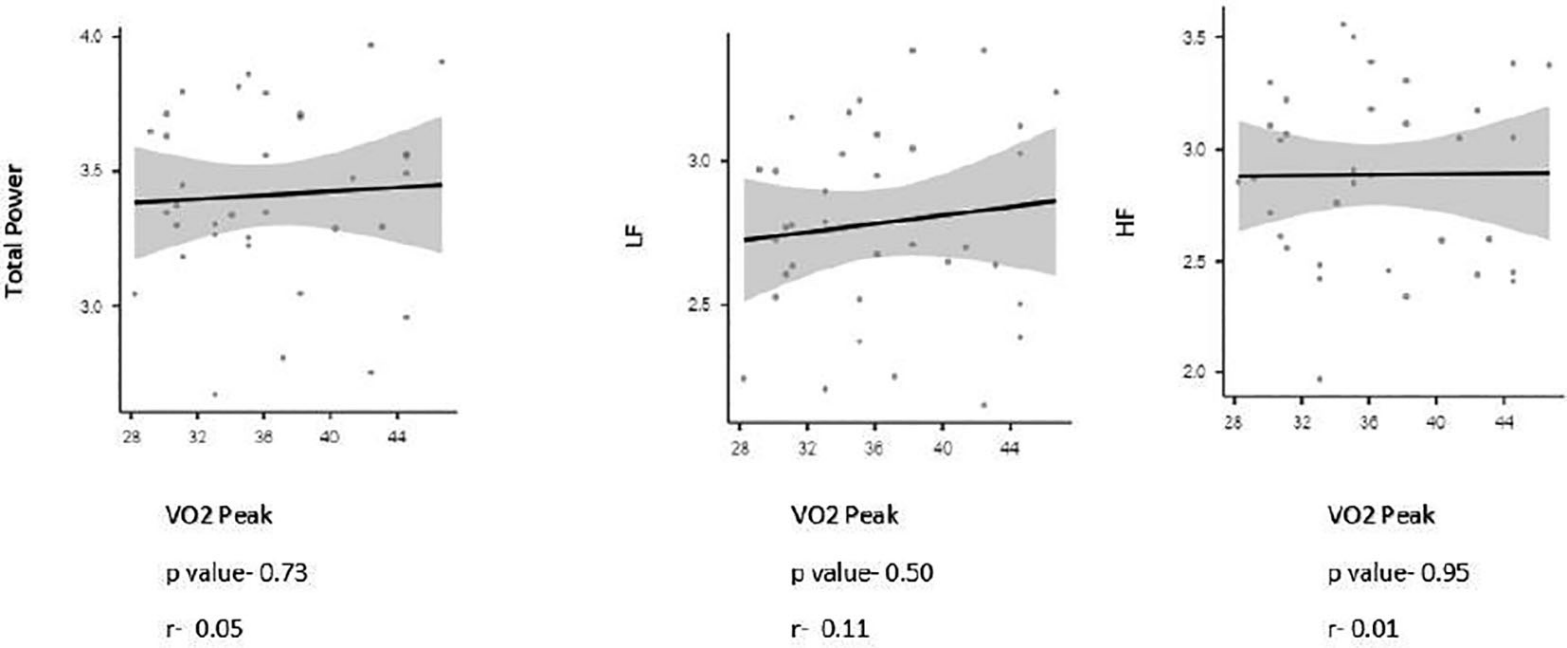
Spearman’s rank correlation coefficient between Heart rate variables and VO _2_ peak.

**Figure 2.**
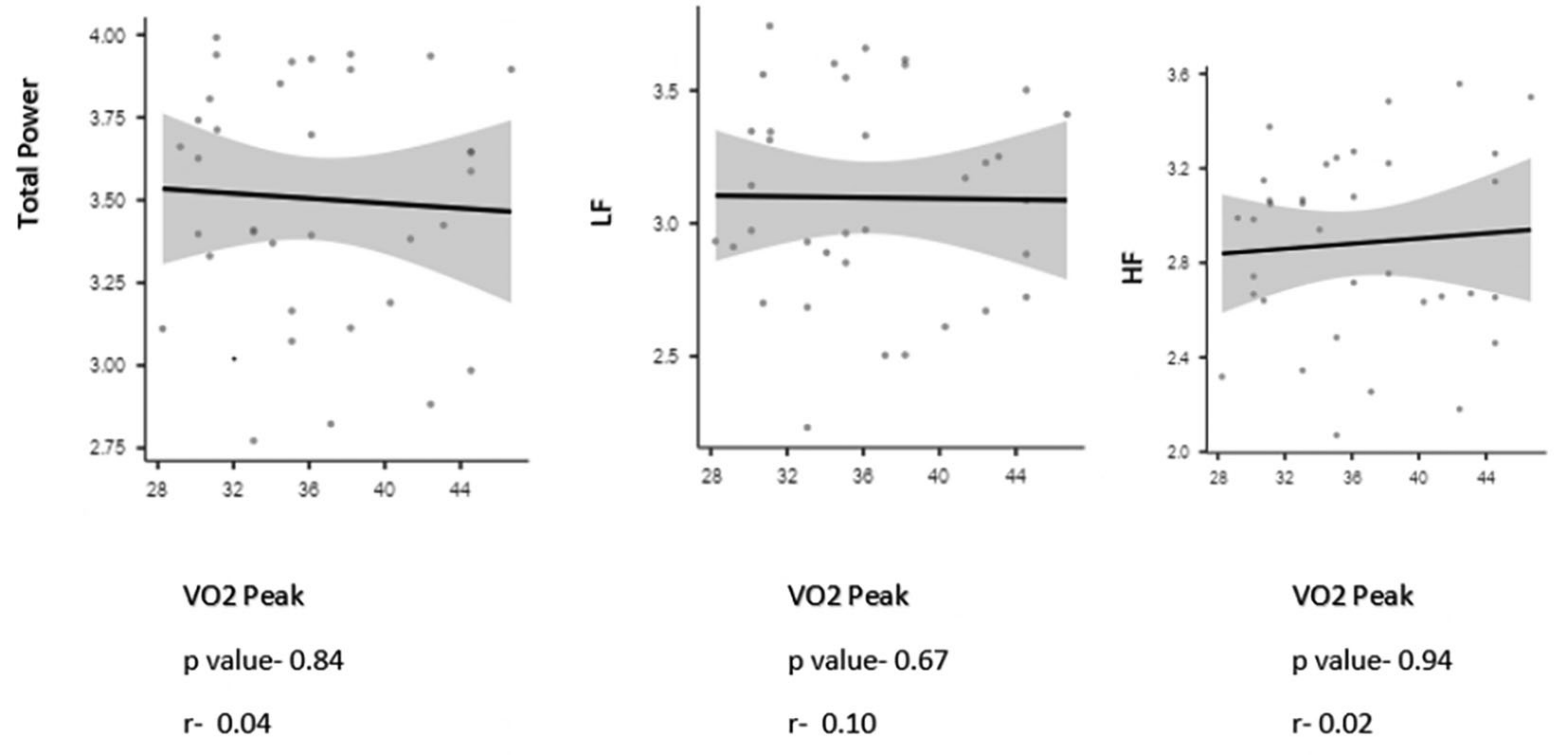
Spearman’s rank correlation coefficient between Pulse rate variables and VO _2_ peak.

**Figure 3.**
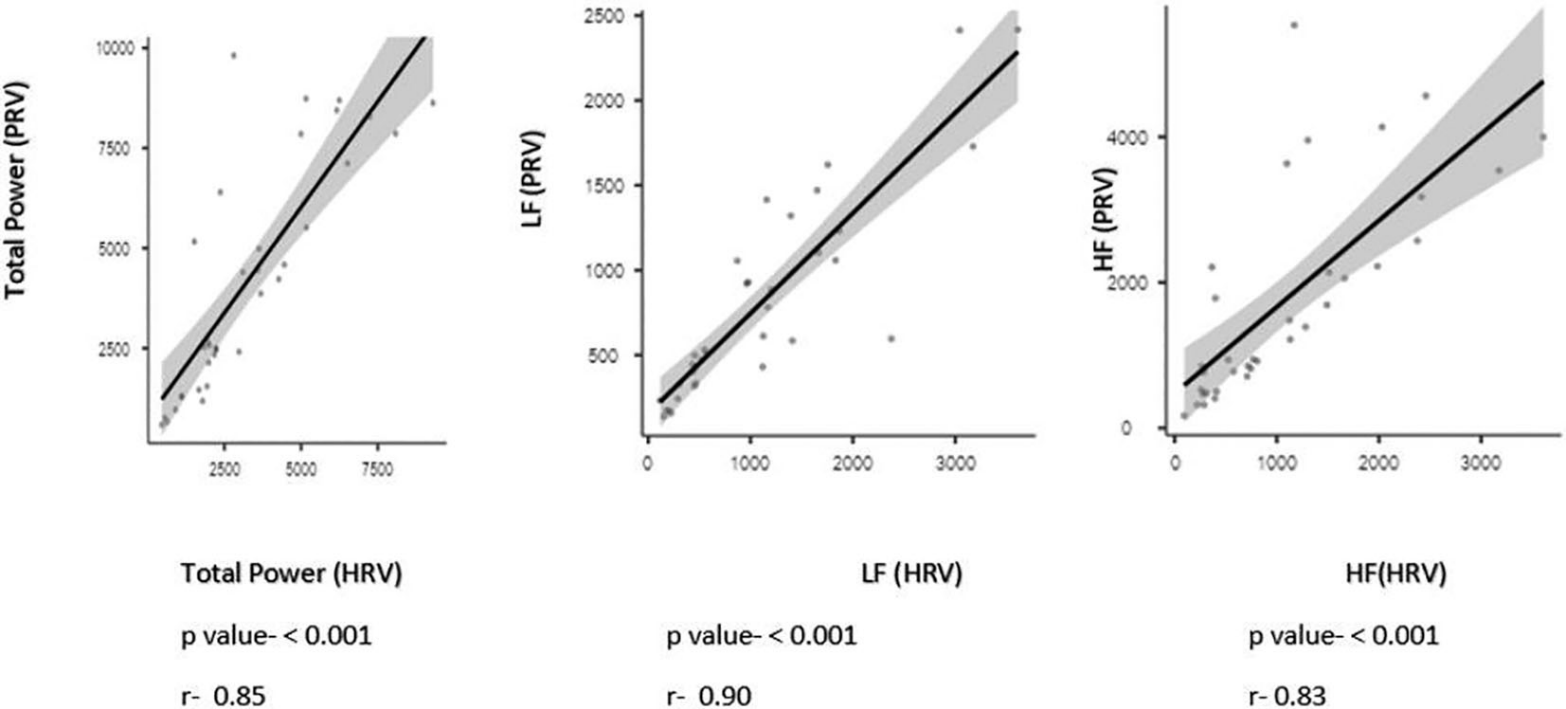
Spearman’s rank correlation coefficient between Pulse rate variables and Heart rate variability.

**Figure 4.**
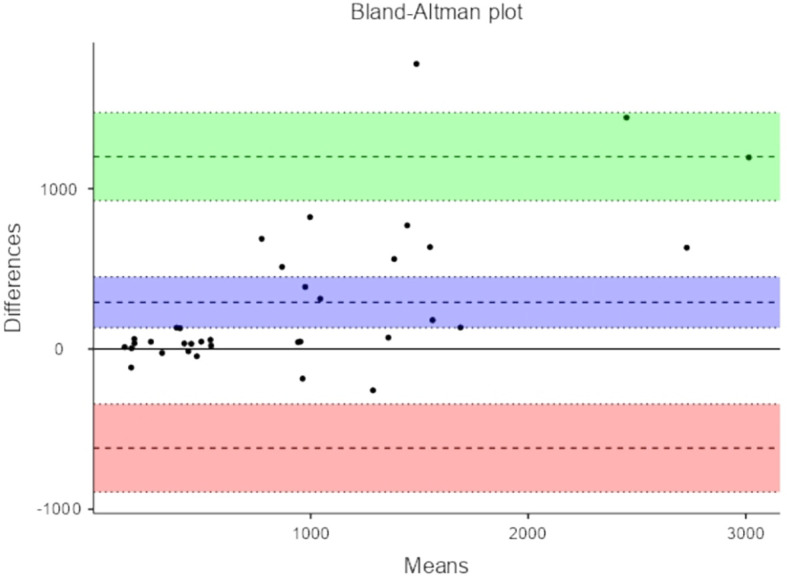
Bland-Altman plots comparing LF measures of Heart rate variability (HRV) and pulse rate variability (PRV). LF - Power in the low-frequency range (0.04–0.15 Hz); HRV - Heart rate variability; PRV - Pulse rate variability.

**Figure 5.**
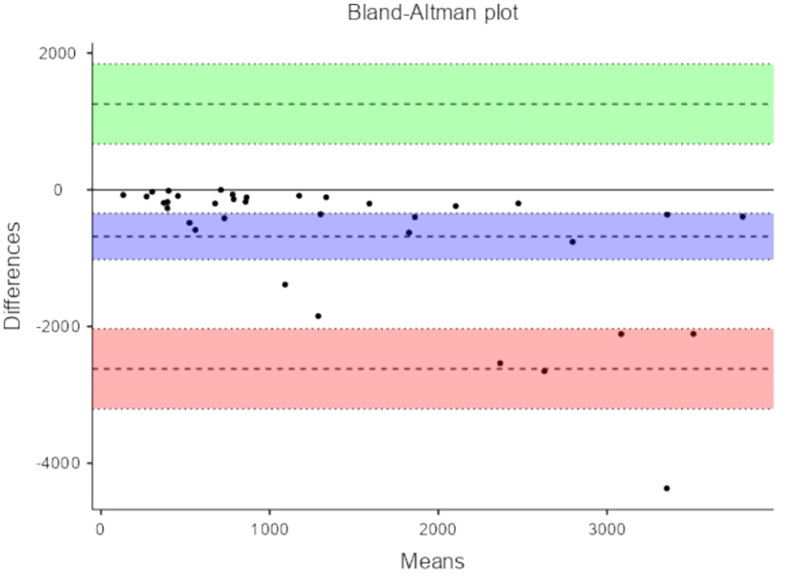
Bland-Altman plots comparing HF measures of Heart rate variability (HRV) and pulse rate variability (PRV). HF - Power in the high-frequency range (0.15–0.4 Hz); HRV - Heart rate variability; PRV - Pulse rate variability.

The study found no significant correlation with the frequency domains variables of HRV/PRV with VO _2_ Peak (Depicted in Figures 1 and 2). The results from the correlation analyses between HRV and PRV are shown in Figure 3. Also Figures 4 and 5 represent Bland-Altman plots comparing HRV and PRV measurements.

## Discussion

The primary objective was agreement analysis, commonly performed using a method like Bland-Altman analysis, assesses the level of agreement or consistency between two measurement methods or variables. It focuses on quantifying the degree of agreement between HRV and PRV by examining the magnitude of the differences between the two measures. The analysis helps determine if the two variables can be used interchangeably or if there are systematic differences or biases between them. In the context of HRV and PRV comparison, agreement analysis investigates the consistency in the measurements of the two variables and whether they can be substituted for one another. In our study Bland-Altman analysis demonstrated the lack of consistency in the measurement of PRV and HRV.

We examined the time-domain measures and frequency-domain metrics of HRV obtained from ECG waveforms and pulse-wave-related HRV (PRV) among overweight individuals. Interestingly, no significant difference were observed between these measures. Additionally, when the HRV and PRV variables were subjected to a log transformation, there were no discernible effects on the study outcomes.

The study revealed that there were no disparities between the HRV and PRV variables, which were concurrently measured for all participants. This suggests that potential confounding factors like age, sex, recording time, temperature, and BMI were similar among the individuals.

Although the study found no statistically significant differences between the HRV and PRV variables, it does not always follow that they are always interchangeable, especially when examining different temperature settings. According to Shin H’s ^21^ study findings, ambient temperature significantly influences PRV compared to HRV, and the difference increases with increasing ambient temperature. The autonomic nervous system, which controls heart rate and affects both HRV and PRV, can be impacted by temperature. ^21^ PRV may contain distinct information that is not available through HRV alone. According to a study report by Mejía-Mejía E, ^10^ PRV reacts to exposure to cold differently than HRV, notably in peripheral areas like the finger and toe, and may contain additional information that HRV does not. In order to evaluate the autonomic activity on many body sites and in various situations, a multi-site PRV assessment is warranted. Further investigations are required to gain a better understanding of the contribution of the sympathetic nervous system to PRV measurements following exposure to varying temperatures.

HRV measures are impacted mainly by heart rate fluctuations brought on by exercise or stress. ^22^ Conversely, PRV reflects the interaction between the heart and peripheral vascular resistance, providing information that HRV does not capture. Different factors cause several significant differences between HRV and PRV. One such distinction is that PRV is assessed by catching the mechanical waves passed from the heart to the fingertips. In contrast, HRV is quantified using the electrical waves recorded in an ECG. ^10^^,^^23^

Changes in temperature can also result in a short lag in the pulse wave’s peak and the R wave on the ECG. Following the electrical current discharge from the sinoatrial (SA) node to the ventricles, the mechanical force produced by the heart’s contraction undergoes substantial attenuation and change as it moves from the left ventricle to the fingertips. In addition, blood viscosity, osmolarity, regulation by the autonomic nervous system, blood vessel size and shape, and individual physical features all have a role in the discrepancy between PRV and HRV. ^24^^,^^25^

The second objective of the current study was to examine the correlation between HRV and PRV. Our main finding displayed that, across all analyzed indices, HRV showed greater associations with PRV. This result is in line with Bulte CS report, 2011. ^26^ The advantage of using pulse wave signals to obtain the PRV is that the technology is less complex and more user-friendly than ECG equipment. Consequently, it can be a suitable option for routine monitoring and serve as an alternative to ECG-based HRV analysis. ^27^

Studies have demonstrated that short recordings of at least 5 minutes can reliably analyze the frequency domain measures of heart rate variability derived from ECG waveform in healthy individuals. ^3^^,^^28^ In our research, we expanded upon previous investigations by employing a broader range of statistical tests, such as mean difference, correlation, and Bland-Altman methods. Additionally, we adhered strictly to comprehensive assessment criteria, thereby enhancing the reliability of our findings. Our results indicate that HRV parameters derived from ECG signals exhibit poor measurement consistency with PRV, but they do exhibit a strong correlation with both sources. Despite the significant correlation between PRV and HRV, caution should be used when substituting PRV with HRV, especially in overweight persons, as inconsistent levels of agreement found in the measurement of PRV and HRV in this study.

## Conclusions

The findings showed no noticeable difference between the median heart rate variability (HRV) and pulse rate variability (PRV). Furthermore, a correlation analysis showed that there was a significant correlation between HRV and PRV. However, there was a poor level of agreement found in the measurement of PRV and HRV. While additional studies are required, it is to shed light on how sympathetic and parasympathetic nervous systems affect PRV. Additionally, PRV measurements from each finger can be compared with HRV.

### Ethics and consent statement

This cross-sectional study is a component of a large intervention trial. This work constitutes a sub-analysis derived from the above-mentioned large intervention trial. The study protocol was approved by the Institutional Research Committee (IRC), and Institutional Ethics Committee of Kasturba Medical College and Kasturba Hospital Manipal (IEC/155/2018). The trial has been registered under the Clinical Trials Registry (CTRI number: CTRI/2018/08/015523). The participants provided their written informed consent to participate in this study.

## Data Availability

Harvard dataverse. Dataset for Heart rate variability and Pulse rate variability analysis, https://doi.org/10.7910/DVN/ONPYF1. ^29^
- Dataset for HRV and PRV.xlsx (This dataset comprises of participant data heart rate variability and pulse rate variability for Total of 35 participants (20 females and 15 males) Harvard dataverse, “Checklist: The Strengthening the Reporting of Observational Studies in Epidemiology (STROBE) Statement: guidelines for reporting observational studies”, https://doi.org/10.7910/DVN/FIXXRS, Harvard Dataverse, V1. ^30^
- STROBE checklist.docx- This file comprises of table depicting The Strengthening the Reporting of Observational Studies in Epidemiology (STROBE) Statement: guidelines for reporting observational studies. Data are available under the terms of the Creative Commons Zero “No rights reserved” data waiver (CC0 1.0 Public domain dedication).
