# Supplementary material for: Analysis of time-domain indices, frequency domain measures of heart rate variability derived from ECG waveform and pulse-wave-related HRV among overweight individuals: an observational study
Source: F1000Res. 2023 Sep 27;12:1229. [Version 1] doi: 10.12688/f1000research.139283.1 (PMC10548108; doi:10.12688/f1000research.139283.1)
Supplement: Peer review history

# Reviewer report: Reviewer response for version 1

**DOI:** 10.5256/f1000research.152548.r210173

**Published:** 2023-10-10

**Authors:** Sanyog Rawat

**Affiliations:** 1 Central University of Rajasthan, Ajmer, Rajasthan, India

- Justify increase or decrease in heart rate variability and pulse rate variability significance in the introduction section.
- Explain how being overweight is going to affect heart rate variability and pulse rate variability.
- Clinical significance may be needed to be strengthened in the discussion section.
- The authors need to highlight several confounders such as diet, sleep, stress in the discussion section.

Is the work clearly and accurately presented and does it cite the current literature?

Yes

If applicable, is the statistical analysis and its interpretation appropriate?

Yes

Are all the source data underlying the results available to ensure full reproducibility?

Yes

Is the study design appropriate and is the work technically sound?

Yes

Are the conclusions drawn adequately supported by the results?

Yes

Are sufficient details of methods and analysis provided to allow replication by others?

Yes

Reviewer Expertise:

Antenna Technology

I confirm that I have read this submission and believe that I have an appropriate level of expertise to confirm that it is of an acceptable scientific standard.

# Reviewer report: Reviewer response for version 1

**DOI:** 10.5256/f1000research.152548.r210176

**Published:** 2023-10-10

**Authors:** Sanjay T Parmar

**Affiliations:** 1 SDM College of Physiotherapy, Dharwad, India

1. Highlight the other cardiac autonomic markers in the introduction.
2. Justify why PRV can serve as a substitute for HRV in the intro section.
3. Add on how adiposity alter the HRV and PRV in intro.

Methods:

 Kindly mention all exclusions.

Discussion:

 Preferably mention log transformed Bland-Altman plots comparing HRV and PRV for log transformed data.

Is the work clearly and accurately presented and does it cite the current literature?

Yes

If applicable, is the statistical analysis and its interpretation appropriate?

Yes

Are all the source data underlying the results available to ensure full reproducibility?

Yes

Is the study design appropriate and is the work technically sound?

Yes

Are the conclusions drawn adequately supported by the results?

Yes

Are sufficient details of methods and analysis provided to allow replication by others?

Yes

Reviewer Expertise:

OVER WEIGHT AND CARDIAC FUNCTION

I confirm that I have read this submission and believe that I have an appropriate level of expertise to confirm that it is of an acceptable scientific standard.

# Reviewer report: Reviewer response for version 1

**DOI:** 10.5256/f1000research.152548.r210175

**Published:** 2023-10-03

**Authors:** Saumya Srivastava

**Affiliations:** 1 Nitte Institute of Physiotherapy, NITTE (Deemed to be University),, Mangalore, Karnataka, India

- Abstract - Result - correlation value should be added along with p value.
- Introduction - can be made elaborate to explain the need for the study.
- Methods - how was the sample size calculated? Mention the validity and reliability of the equipment and tools used in the study.
- Discussion - can be backed with a few more studies and reasoning.

Is the work clearly and accurately presented and does it cite the current literature?

Yes

If applicable, is the statistical analysis and its interpretation appropriate?

I cannot comment. A qualified statistician is required.

Are all the source data underlying the results available to ensure full reproducibility?

Yes

Is the study design appropriate and is the work technically sound?

Yes

Are the conclusions drawn adequately supported by the results?

Yes

Are sufficient details of methods and analysis provided to allow replication by others?

Yes

Reviewer Expertise:

Musculoskeletal conditions, LBP, Sacroiliac dysfunctions

I confirm that I have read this submission and believe that I have an appropriate level of expertise to confirm that it is of an acceptable scientific standard.

# Reviewer report: Reviewer response for version 1

**DOI:** 10.5256/f1000research.152548.r210177

**Published:** 2023-10-03

**Authors:** Aishwarya Nair

**Affiliations:** 1 Nitte Institute of Physiotherapy, NITTE (Deemed to be University),, Mangaluru, Karnataka, India

The article is written very well.

 In Introduction, the time domain indices, and frequency domain measurements may be elaborated on more so that therapists of various professional grade may understand and apply. 

 In Participant recruitment, kindly provide reference for the sentence regarding abstinence from alcohol and caffeine. Why these two drinks are considered above others, can be clarified.

Is the work clearly and accurately presented and does it cite the current literature?

Yes

If applicable, is the statistical analysis and its interpretation appropriate?

Yes

Are all the source data underlying the results available to ensure full reproducibility?

Yes

Is the study design appropriate and is the work technically sound?

Yes

Are the conclusions drawn adequately supported by the results?

Yes

Are sufficient details of methods and analysis provided to allow replication by others?

Yes

Reviewer Expertise:

Pulmonary Rehabilitation, Cardiac Rehabilitation, Critical care, neonates, pediatric

I confirm that I have read this submission and believe that I have an appropriate level of expertise to confirm that it is of an acceptable scientific standard.
